# To grow old: regulatory role of ethylene and jasmonic acid in senescence

**DOI:** 10.3389/fpls.2015.00020

**Published:** 2015-01-29

**Authors:** Joonyup Kim, Caren Chang, Mark L. Tucker

**Affiliations:** ^1^Soybean Genomics and Improvement Laboratory, United States Department of Agriculture–Agricultural Research Service, Beltsville, MD, USA; ^2^Department of Cell Biology and Molecular Genetics, University of Maryland, College Park, MD, USA

**Keywords:** ethylene, jasmonic acid, leaf senescence, cross-talk, miRNA, transcription factors

## Abstract

Senescence, the final stage in the development of an organ or whole plant, is a genetically programmed process controlled by developmental and environmental signals. Age-related signals underlie the onset of senescence in specific organs (leaf, flower, and fruit) as well as the whole plant (monocarpic senescence). Rudimentary to most senescence processes is the plant hormone ethylene, a small gaseous molecule critical to diverse processes throughout the life of the plant. The role of ethylene in senescence was discovered almost 100 years ago, but the molecular mechanisms by which ethylene regulates senescence have been deciphered more recently primarily through genetic and molecular studies in *Arabidopsis*. Jasmonic acid (JA), another plant hormone, is emerging as a key player in the control of senescence. The regulatory network of ethylene and JA involves the integration of transcription factors, microRNAs, and other hormones. In this review, we summarize the current understanding of ethylene’s role in senescence, and discuss the interplay of ethylene with JA in the regulation of senescence.

## INTRODUCTION

Senescence, the cessation of growth in cells, organs or the whole plant, is a highly regulated developmental process affected by environmental factors (Lim et al., 2007; Guo and Gan, 2012; Li et al., 2012). It is a pivotal turning point in plant growth and development that most often terminates with a process termed programmed cell death (PCD). Senescence (and PCD) is an active degenerative process linked to physiological and biochemical changes at the cellular, organ, and whole plant level. For example, senescence of photosynthetic tissues (e.g., leaves, stems) is accompanied by a gradual de-greening process exhibited by the loss of chlorophyll and thylakoid membranes, and increase in lipid-containing plastoglobuli (Hensel et al., 1993; Grbić and Bleecker, 1995; Gepstein, 2004). In *Arabidopsis*, the transition of growth to senescence constitutes the conversion from maintaining somatic tissues to supporting reproductive development by rebalancing the allocation of plant resources (Gan and Amasino, 1997; Guo and Gan, 2005; Lim et al., 2007). Transcriptome analyses of several plant species reinforce earlier observations that many genes are differentially regulated during senescence (Garbarino and Belknap, 1994; Genschik et al., 1994; Nam, 1997; Guo et al., 2004; Breeze et al., 2011). Large-scale gene expression studies highlight changes in key regulatory components including receptor-like kinases (e.g., SARK and SIRK), transcription factors (e.g., WRKY, NAC), mitogen activated protein kinases (MAPKs), metabolic pathway, and signaling pathway components for plant hormones and stress responses (Guo et al., 2004; Breeze et al., 2011). Although senescence in polycarpic and monocarpic plants may share different outcomes, it is generally accepted that the major role for senescence is the recycling of nutrients and stored energy from somatic tissues to young developing organs and reproductive organs.

The plant hormone ethylene is critical to a diverse set of developmental programs and both abiotic and biotic stress responses. In addition, ethylene affects plant aging programs such as monocarpic senescence of the whole plant as well as individual leaves, flowers, and fruit (i.e., ripening; Abeles et al., 1992). Although the involvement of ethylene in senescence has been known for over 100 years, components in the ethylene-signaling pathway and the mechanisms of action were more recently identified mostly through work in *Arabidopsis*.

Jasmonic acid (JA) plays a major role in plant defense as well as growth and development, including leaf and reproductive organs. Identification of JA as a plant hormone signal occurred much more recently than that of ethylene (Sembdner and Parthier, 1993). One of the first reports of JA as a hormone was the identification of methyl-jasmonate (meJA) as the senescence-promoting substance in wormwood (Ueda and Kato, 1980). Studies in *Arabidopsis* indicate that JA is associated with the timing of senescence programs in both somatic tissues and reproductive organs (He et al., 2002; Castillo and Leon, 2008; Schommer et al., 2008; Danisman et al., 2012; Kim et al., 2013a). Notably, recent studies revealed that JA’s role in senescence involves similar regulatory circuits as used by ethylene [e.g., microRNA (miRNA)-transcription factors; Schommer et al., 2008]. In summary, the published results indicate that cross-talk between JA and ethylene fine-tunes the onset and timing of senescence in *Arabidopsis*.

Our objective here is to summarize current advances in understanding the mechanism by which ethylene regulates leaf senescence in *Arabidopsis*, and the role of JA in senescence in the context of its interplay with ethylene.

## ETHYLENE AND LEAF SENESCENCE

All major hormones are found to be associated with leaf senescence either positively or negatively (Gan and Amasino, 1997; Khan et al., 2014). Although ethylene was one of the first plant hormones identified to accelerate senescence (Neljubow, 1901; Crocker, 1932), the mechanism by which ethylene regulates senescence remained inexplicit. Recent genetic and molecular studies in *Arabidopsis* have unraveled a fundamental signaling pathway for ethylene based on the ethylene regulated growth response (triple response) of etiolated seedlings. Briefly, ethylene is first perceived by its receptor [e.g., ETHYLENE RESPONSE 1 (ETR1)] residing at the endoplasmic reticulum (ER) membrane. Upon binding ethylene, the receptor stops activating the serine/threonine protein kinase CONSTITUTIVE RESPONSE 1 (CTR1), which causes CTR1 to stop phosphorylating the ER membrane-localized ETHYLENE INSENSITIVE 2 (EIN2) protein. When not phosphorylated, the cytosolic C-terminus of EIN2 is cleaved by an unidentified protease. After cleavage, the EIN2 C-terminus moves into the nucleus where it activates transcription (Ju et al., 2012; Qiao et al., 2012; Wen et al., 2012; Ji and Guo, 2013). ETHYLENE INSENSITIVE 3 (EIN3) and its close homolog, ETHYLENE INSENSITIVE3-LIKE 1 (EIL1), are key transcription factors activated by EIN2 (Alonso et al., 2003; Guo and Ecker, 2003; Potuschak et al., 2003; Binder et al., 2004, 2007; Gagne et al., 2004; An et al., 2010).

Much of what we currently know about the role of ethylene signaling in senescence comes from studies with genetic mutants that lack the ethylene dependent triple response in dark-grown seedlings. For example, the *Arabidopsis* ethylene-insensitive mutants *etr1-1*, *ein2-1*, and *ein1-1* (which is allelic to *etr1-1*) were shown to have a delayed onset of leaf senescence and expression of leaf senescence marker genes, e.g., *SAG1*, *SAG2*, and *SAG12* (Grbić and Bleecker, 1995). The leaves of these mutants do, however, eventually senesce indicating that the role for ethylene in leaf senescence is not essential, but, in *Arabidopsis*, modulates the timing of senescence. In support of this role, when ethylene perception or biosynthesis was genetically inhibited in tobacco and tomato, the plants exhibited a delay in the onset of leaf senescence (Picton et al., 1993; John et al., 1995; Yang et al., 2008). In contrast, mutants with enhanced ethylene biosynthesis or a constitutive ethylene response (e.g., *ctr1*) did not always display an early onset of senescence (Guzman and Ecker, 1990; Kieber et al., 1993; Lanahan et al., 1994; Woeste et al., 1999). For instance, the *ctr1* mutant of *Arabidopsis*, which possesses constitutively activated ethylene signaling, did not display precocious leaf senescence, and the exogenous application of ethylene to the mutant did not affect the rate of senescence (Kieber et al., 1993). The lack of precocious leaf senescence in the *ctr1* mutant may be partly due to the fact that the *ctr1* plants display a delayed flowering time compared to wild-type plants (Hua and Meyerowitz, 1998; Hall and Bleecker, 2003); however, when leaves were detached from the *ctr1* mutant, the detached leaves clearly displayed accelerated senescence (Xu et al., 2014). Moreover, ectopic expression of 1-aminocyclopropane-1-carboxylic acid (ACC) synthase in tomato, which overproduces ethylene, did not accelerate the rate of leaf senescence (Lanahan et al., 1994). These results suggest that, although ethylene plays a role in the timing of senescence, there are developmental conditions including flowering and/or other environmental cues that must precede ethylene if ethylene is to induce a senescence response.

Additional support for the role of ethylene in senescence comes from the identification of *Arabidopsis* mutants with a delayed leaf senescence phenotype (Oh et al., 1997). The *oresara* mutants (*ore1*, *ore2*, *ore3*, and *ore9*) were identified as having a decrease in chlorophyll content, an indication of leaf senescence, and reduced photochemical efficiency of PSII (Fv/Fm), a parameter of functional leaf senescence (Oh et al., 1997). Genetic complementation of *ore2* and *ore3* confirmed that these two mutants are allelic to *ein2*, supporting earlier observations that the signaling cascade of ethylene is tightly associated with leaf senescence.

Between 2004 and 2005, several laboratories independently demonstrated that the miRNA, miR164, is involved in the control of mRNA levels for NAC-domain transcription factors, which were previously shown to be essential for organ differentiation and development (Laufs et al., 2004; Mallory et al., 2004; Baker et al., 2005; Guo et al., 2005). In 2009, Kim et al. (2009) reported that the *ORE1*, which was identified earlier in a screen for senescence associated genes (*SAGs*; Woo et al., 2004), is allelic to *NAC2*; moreover, they demonstrated that *miR164ABC* expression inversely correlated with the expression of *NAC2* transcript. Through a combination of overexpression lines and genetically suppressed mutants of *miR164*, *NAC2* and *EIN2*, they demonstrated a link between leaf senescence, ethylene signaling, and a decline in miR164 RNA, which leads to an increase in *NAC2* expression (Kim et al., 2009; Li et al., 2013).

The role of ethylene signaling was further substantiated by the identification of *EIN3* as another *SAG* in the ethylene-induced leaf senescence network and that elevated EIN3 expression accelerated the onset of senescence (Li et al., 2013). In addition, Li et al. (2013) demonstrated by chromatin immunoprecipitation (ChIP) and electrophoretic mobility shift assays (EMSA) that EIN3 binds to the promoter for *miR164*. Subsequently, it was demonstrated by ChIP and a yeast one-hybrid (Y1H) assay that EIN3 not only binds to the *miR164* promoter but also directly binds to the promoters of *NAC2* and another NAC-domain gene, *NAP*, both of which were identified as *SAGs* (Kim et al., 2014). EIN3 and EIL1 are closely related homologues that appear to have redundant and distinct functions in ethylene responses (Chao et al., 1997; Binder et al., 2007). Both Li et al. (2013) and Kim et al. (2014) used *ein3 eil1* double mutants to preclude complications in interpretation that might arise from their functional redundancy. They observed that *ein2* mutants displayed a more delayed senescence phenotype than the *ein3 eil1* double mutants. They concluded that EIN2, which precedes EIN3 and EIL1 in the ethylene-signaling pathway, controls senescence that is both dependent and independent of EIN3/EIL1. They proposed that EIN2 signaling somehow bypasses EIN3/EIL1. Although both Li et al. (2013) and Kim et al. (2014) suggested that EIN2–EIN3–miR164 regulate the timing of leaf senescence, further experiments may be necessary to firmly establish EIN3’s role in the natural progression of leaf senescence. For instance, it may be useful to examine the expression of other EIL proteins (i.e., EIL2, EIL3, and EIL4) in relation to EIN3, particularly in leaf senescence.

To summarize, the proposed role for ethylene signaling in leaf senescence is to coordinate the timing of leaf senescence by integrating miR164-transcription factors in concert with environmental and age-related signals, which appears to ensure the timely and efficient transition from an active photosynthetic organ to a degenerating organ and to also salvage nutrients for development of reproductive organs and young leaves (Grbić and Bleecker, 1995).

## JASMONIC ACID AND LEAF SENESCENCE

Jasmonic acid is another plant hormone that modulates defense responses, growth and development, and is also proposed to mediate leaf senescence (He et al., 2002; Schommer et al., 2008; Shan et al., 2011). A review of the literature on the involvement of JA in leaf senescence is, however, sometimes contradictory (Taylor and Whitelaw, 2001; Seltmann et al., 2010a,b) and not fully resolved. For example, many JA biosynthesis genes are differentially regulated, as some are up-regulated and others are down-regulated in the progression of leaf senescence (He et al., 2002). In addition, several studies suggested that synthesis of JA during leaf senescence might be a secondary byproduct from the breakdown of macromolecules and membranes in the process of senescence. These studies suggested that the increase in oxylipins levels such as JA and 12-oxo-phytodienoic acid (OPDA) do not necessarily indicate a role for JA in natural leaf senescence but a byproduct of senescence (Seltmann et al., 2010a,b).

However, in *Arabidopsis*, recent studies suggest that JA does have a role in leaf senescence. Mutations in the JA receptor and JA biosynthesis genes, and physiological responses to JA or various JA derivatives, indicate that JA does mediate the timing of leaf senescence. *Arabidopsis* mutants *allene oxide synthase* (*aos*), *oxophytodienoate-reductase 3* (*opr3*), which have reduced levels of JA, and *coronatine insensitive 1* (*coi1*), which is insensitive to JA, exhibit temporal shifts in the onset of natural and dark-induced senescence (He et al., 2002; Castillo and Leon, 2008; Schommer et al., 2008; Danisman et al., 2012). In addition, exogenous application of JA on wild-type *Arabidopsis* promotes leaf senescence and induces the expression of several *SAGs*, including JA biosynthesis genes (He et al., 2002). Moreover, in naturally senescing leaves, a gradual increase in the transcript levels of JA biosynthesis genes (e.g., *AOS*, *LIPOXYGENASE1* (*LOX1*), *LOX3*, *LOX4*, *OPR1*, and *OPR3*) was observed (He et al., 2002). Thus, results with *Arabidopsis* support earlier proposals that JA positively affects the timing of leaf senescence in conjunction with other age-related cues (He et al., 2002).

Recently, it was shown that the miRNA miR319 [*JAGGED AND WAVY* (*JAW*)] regulates the mRNA levels of the transcription factors called TEOSINTE BRANCHED/CYCLOIDEA/PCF (TCP; Schommer et al., 2008). TCPs are plant-specific transcription factors identified as negative regulators of plant growth and development (Almeida et al., 1997; Doebley et al., 1997; Luo et al., 1999). In addition, TCPs were found to be critical for the expression of cell cycle regulators called proliferating cell nuclear antigen, PCNA (Kosugi and Ohashi, 1997). Schommer et al. (2008) showed that miR319, which affected the expression of class II TCP2/4/10, altered the expression of JA biosynthesis genes and the endogenous levels of JA. More specifically, they demonstrated that the miR319-TCP regulatory module regulated JA biosynthesis by repressing the expression of *LOX2*. They also showed that the reduced levels of JA resulted in a delay in leaf senescence, and that the delayed senescence phenotype was reversed by application of JA. They concluded that, whereas TCP2/4/10 negatively regulate leaf growth, they positively regulate leaf senescence.

In a separate study, it was revealed that a class I TCP (TCP20) also regulates *LOX2* (Danisman et al., 2012). Mutations in *TCP20* caused early onset of leaf senescence indicating that TCP20 acts as a negative regulator. They further demonstrated that TCP20 binds to the promoter of the *LOX2* gene (Danisman et al., 2012). They proposed that class I and class II TCPs act antagonistically to regulate *LOX* gene expression. Interestingly, RNAi suppression of *LOX2* expression, which was targeted specifically to mature leaves by using the *SAG13* promoter, greatly decreased the accumulation of JA as leaves aged naturally but had no effect on chlorophyll loss (Seltmann et al., 2010b). Conversely, when senescence was induced prematurely by treatment with sorbitol, chlorophyll loss and *SEN1* and *SAG13* expression were delayed. However, it is worth noting that the peak transcript levels of *LOX2* preceded the onset of leaf senescence and declined as leaf senescence progressed (He et al., 2002), as reflected in a decline in the level of JA (Seltmann et al., 2010b). It is possible that the regulation of miR319-TCP-*LOX2* represents one of the early regulatory mechanisms in leaf development that alters leaf senescence and that expression of *LOX2*-RNAi with the *SAG13* promoter occurred too late in the natural aging process to yield a delayed senescence response. In summary, although some results for JA signaling seem to contradict a role for JA in leaf senescence, it is worth emphasizing that the phenotype of the *miR319* mutant, *jaw-*D, could be reversed by the exogenous application of meJA (Schommer et al., 2008).

## INTERPLAY BETWEEN ETHYLENE AND JA IN LEAF SENESCENCE

Although the interaction between ethylene and JA has been well studied in diverse developmental, abiotic and biotic responses (Turner et al., 2002; Lorenzo et al., 2003; Memelink, 2009; Zhu et al., 2011; Kim et al., 2013a; Song et al., 2014; Zhang et al., 2014), a clear understanding of the interplay between ethylene and JA in mediation of leaf senescence is less well defined. Nevertheless, recent work on ethylene and JA signaling is beginning to shed light on the regulatory mechanisms by which these two senescence-affecting hormones control the timing of leaf senescence.

Several studies have defined mechanisms by which ethylene and JA interact to regulate plant development and pathogen defense (Zhu et al., 2011; Song et al., 2014; Zhang et al., 2014). Zhu et al. (2011) showed that EIN3 and EIL1 positively regulate JA-mediated responses such as root hair development, resistance to necrotrophic pathogens, and related gene expression. They demonstrated that JAZ proteins (e.g., JAZ1, 3, and 9) physically interact with EIN3 and EIL1, and repress the transcriptional activities of EIN3/EIL1. They further showed that JAZ protein (JAZ1) recruits HISTONE DEACETYLASE 6 (HDA6), which deacetylates the chromatin of EIN3/EIL1 and represses EIL3/EIL1-dependent transcription and JA signaling. It would be interesting to know if a similar mechanism is utilized in senescence. In this regard, it has been demonstrated that HDA6 positively regulates JA-mediated leaf senescence as well as flowering time, which also influences the timing of senescence (Wu et al., 2008).

In addition to HDA6 control of EIN3/EIL1, Song et al. (2014) demonstrated that JA activated MYC2 repressed EIN3/EIL1 transcription. Conversely, EIN3/EIL1 repressed MYC2, which in turn inhibits JA responses. This reciprocating regulation may fine-tune ethylene/JA responses. Specifically, they showed that MYC2 represses transcriptional activities of EIN3/EIL1 to attenuate the mRNA levels of *HOOKLESS 1* (*HLS1*, a positive regulator of apical hook development) and *ETHYLENE RESPONSE FACTOR 1* (*ERF1*), which thereby inhibits ethylene-induced apical hook curvature. In addition, they showed that EIN3/EIL1 interact with MYC2/3/4 to attenuate JA-induced expression of wound—herbivory—responsive genes, which reduces JA-regulated defense against herbivores.

In another independent study, Zhang et al. (2014) also demonstrated an antagonistic regulation between MYC2 and EIN3 in the ethylene-induced hook curvature formation. In this study, they showed that JA inhibits the formation of apical hook curvature by reducing *HLS1* gene expression. They showed that JA-activated MYC2 transcriptionally represses *EIN3* to reduce *HLS1* activity. They proposed a dual mode of action for MYC2. Firstly, MYC2 promotes expression of the *EIN3 BINDING F-BOX PROTEIN 1* (*EBF1*) that then promotes EIN3 degradation and, secondly, MYC2 repression of EIN3 inhibits the positive regulation by EIN3 of *HSL1*. However, it remains to be determined if these mutual antagonistic actions between ethylene and JA signaling in the apical hook formation also regulate the timing of leaf senescence.

More specific to senescence are two studies by Li et al. (2013) and Kim et al. (2013a). As discussed earlier, Li et al. (2013) demonstrated that JA regulation of senescence is dependent upon the key ethylene-signaling components EIN2 and EIN3/EIL1. In comparison to wild-type leaves, detached leaves of *ein2* and *ein3 eil1* mutants treated with 50 μM of meJA largely stayed green. In addition, when wild-type leaves were co-treated with 100 μM silver nitrate (AgNO3), an inhibitor of ethylene action, and 50 μM of meJA, the leaves remained largely green, suggesting that JA-induced leaf senescence is dependent upon ethylene signaling (Li et al., 2013).

**FIGURE 1:**
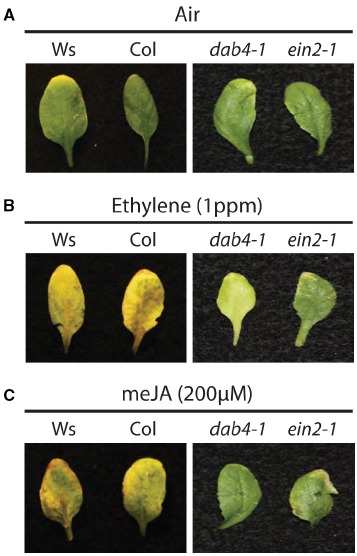
**Interdependency of ethylene and JA in leaf senescence.** Leaf senescence phenotypes with air **(A)**, ethylene **(B)**, and meJA **(C)** treatments in wild-type plants (Ws and Col), a JA receptor mutant (*dab4-1/coi1-37*) and an ethylene insensitive mutant (*ein2-1*). This figure was originally published in Kim et al. (2013a) as supplemental Figure S4. Whole plant assays were carried out in a closed chamber 6 weeks after germination when the plants had bolted as previously described (Kim et al., 2013a,b).

In another project, Kim et al. (2013a) examined the interdependency of ethylene and JA in floral organ abscission. Although leaf senescence was not a major focus of that report, they did collect data for leaf senescence as well. In that project they observed that exogenous application of 200 μM meJA accelerated the timing of floral organ abscission and leaf senescence in the *ein2-1* mutant (Figure 1). Kim et al. (2013a) proposed that ethylene and JA act partly in parallel pathways to regulate the timing of both floral organ abscission and leaf senescence. Although the degree of accelerated leaf senescence in the Kim et al. (2013a) study appears to be minor, the discrepancy of JA dependency on EIN2 in the two separate senescence studies may be explained by differences in how the experiments were performed. In the Li et al. (2013) study, the leaves were detached from the wild type and *ein2-5* plants at 3–4 weeks and then treated with a low concentration of 50 μM meJA. In the Kim et al. (2013a) study, after 6 weeks when the plants had bolted and produced several inflorescences, the whole plant was sprayed twice a day for 3 days with 200 μM meJA. In addition to the different meJA concentrations, which may have had a minor influence, the developmental stage of the leaves were different and, as previously mentioned, the development stage of the plant is also important to how JA influences the timing of senescence (Kim et al., 2013b; Li et al., 2013).

**FIGURE 2:**
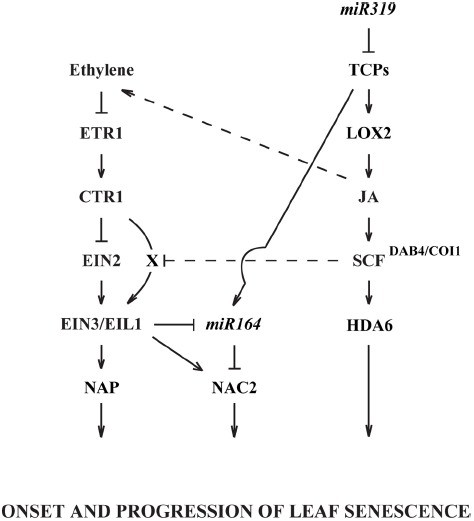
**A schematic diagram for interactions between ethylene and JA in the regulation of leaf senescence.** Known regulations are shown in solid lines and unidentified pathways are represented in dotted lines. X denotes an unknown ethylene-signaling component that may be inhibited by JA or SCF^DAB4/COI1^. See the text for details and supporting references.

Of further interest in regard to ethylene and JA interdependency is that JA was found to alter ethylene signaling downstream of the ethylene receptors (Kim et al., 2013b). In this study, it was discovered that when JA synthesis was chemically or genetically inhibited with phenidone or *dde2-2*, respectively, *ein2-1* mutants abscised earlier, and root growth was inhibited in responsive to 1 μL L^–1^ ethylene. Similar results were obtained with the *coi1 ein2* double mutants. They concluded that there is an EIN2-independent pathway for ethylene signaling that is inhibited by JA and, when JA is very low or JA signaling is blocked as in the *coi1* mutant, the EIN2 independent ethylene signaling pathway is discernible in the *ein2* mutant. In this study, the authors did not examine leaf senescence. It would, however, be interesting to know if the EIN2 independent ethylene signaling pathway plays a role in leaf senescence and JA responsiveness (Figure 2).

## CONCLUDING REMARKS AND PERSPECTIVE

Recent technological advances in transcriptome analyses, including mRNA and miRNA sequencing and ChIP, have greatly enhanced our understanding of signaling networks that control developmentally and environmentally affected process. Although these tools have helped to significantly advance our understanding of how ethylene and JA regulate senescence, there is still much more to learn. We have reviewed studies demonstrating the role of ethylene perception and its signaling pathway in the regulation of senescence. Of particular interest were recent findings demonstrating the interaction between miR164 and EIN3 to regulate the expression of *NAC2*, which in turn regulates the onset of senescence (Figure 2). We also reviewed independent studies of JA’s control of senescence and how miR319 regulates the mRNA level of class II TCPs, which antagonistically work with class I TCPs to control the expression of *LOX2*, which is needed for synthesis of JA (Figure 2). In addition, we briefly reviewed the role of HDA6 and MYC2 in other developmental and defense responses where ethylene and JA signaling interact.

Senescence and the timing of the onset of senescence are complex. There is evidence to suggest that all of the known plant hormones have some influence over the timing of abscission, and many environmental factors and additional internal cues modulate this process (Gan and Amasino, 1997; Khan et al., 2014). Discovery of miRNAs and transcription factors involved in the regulation of the onset of leaf senescence identifies additional points in the network of senescence signals by which the complex process of senescence can be modulated by environmental and internal cues.

In addition to what we currently know about the regulation of leaf senescence, basic questions still need to be addressed. JA control of senescence appears to be dependent on ethylene perception and signaling except for a nuance, which suggests that at low JA levels ethylene signaling can bypass EIN2 (Kim et al., 2013b). Although ethylene signaling may bypass EIN2 under certain conditions, ethylene perception was shown to be necessary for JA responsiveness. It seems logical that JA might modulate senescence through regulation of ethylene biosynthesis (Figure 2). In fact, JA regulation of ethylene biosynthesis has been proposed for other processes (Wang et al., 2002), but the mechanism of regulation has not been well established. ACC is the immediate precursor to ethylene, and Staswick and Tiryaki (2004) discovered that JA can form a conjugate with ACC (JA-ACC). These authors suggested that conjugation of ACC by JA might play a role in regulating ethylene biosynthesis.

Also of interest are studies on *miR319* and TCPs. It was demonstrated that *miR319* and TCPs regulate *CUC2* (*NAC2*/*ORE1*) expression during meristem development (Koyama et al., 2007). This work on regulation of morphogenesis in the apical meristem suggests that *NAC2* expression during senescence may also be regulated by miR319 and TCPs during leaf senescence. If true, this would tie together regulation of JA biosynthesis and ethylene signaling through regulation of *EIN3* and *NAC2* (Figure 2). The role of HDA6 and MYC2 in regulating ethylene and JA interactions in the control of apical hook formation and defense responses is interesting and should be examined further for a regulatory role in senescence. Much still needs to be done to understand the multitude of factors that mediate aging and the timing of senescence in both plant organs and whole plants.

### Conflict of Interest Statement

The authors declare that the research was conducted in the absence of any commercial or financial relationships that could be construed as a potential conflict of interest.
